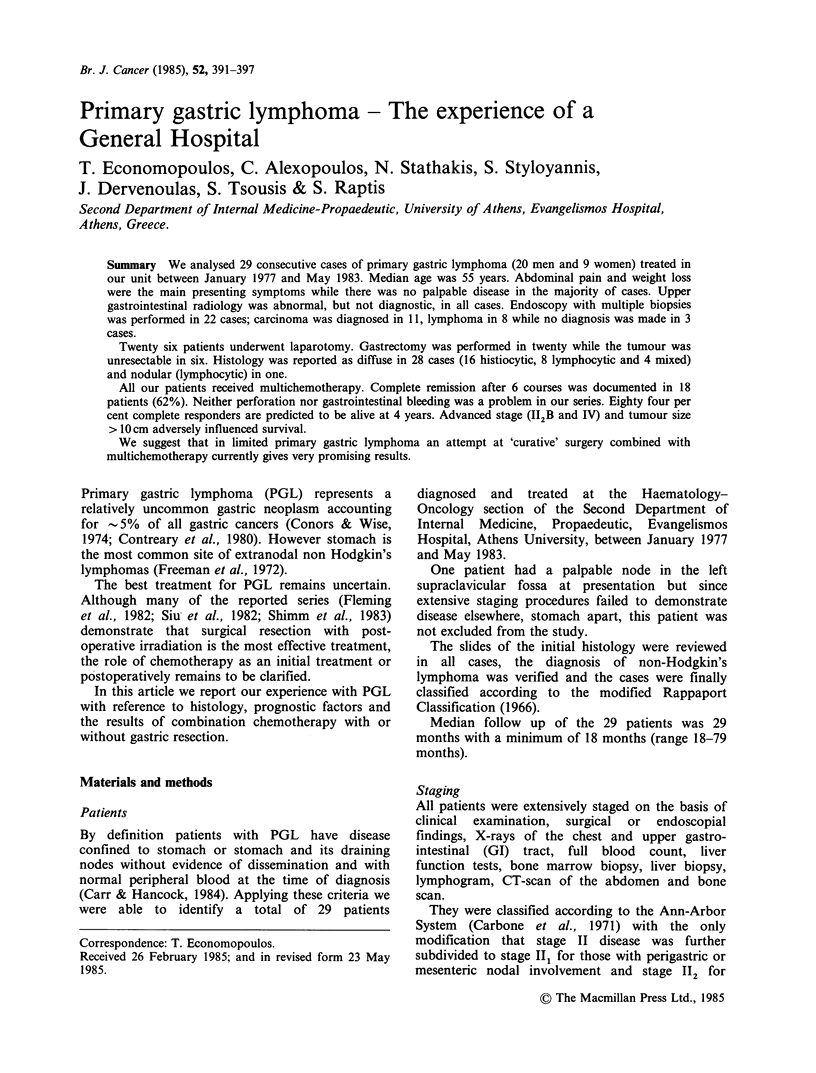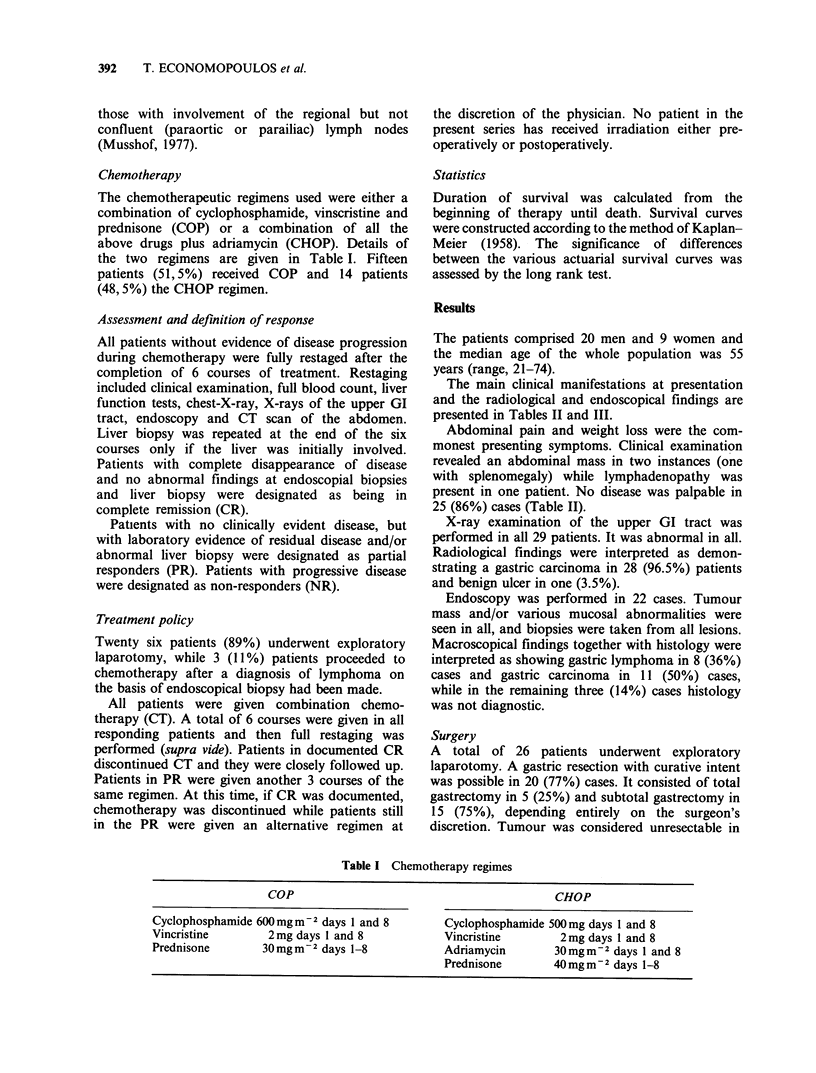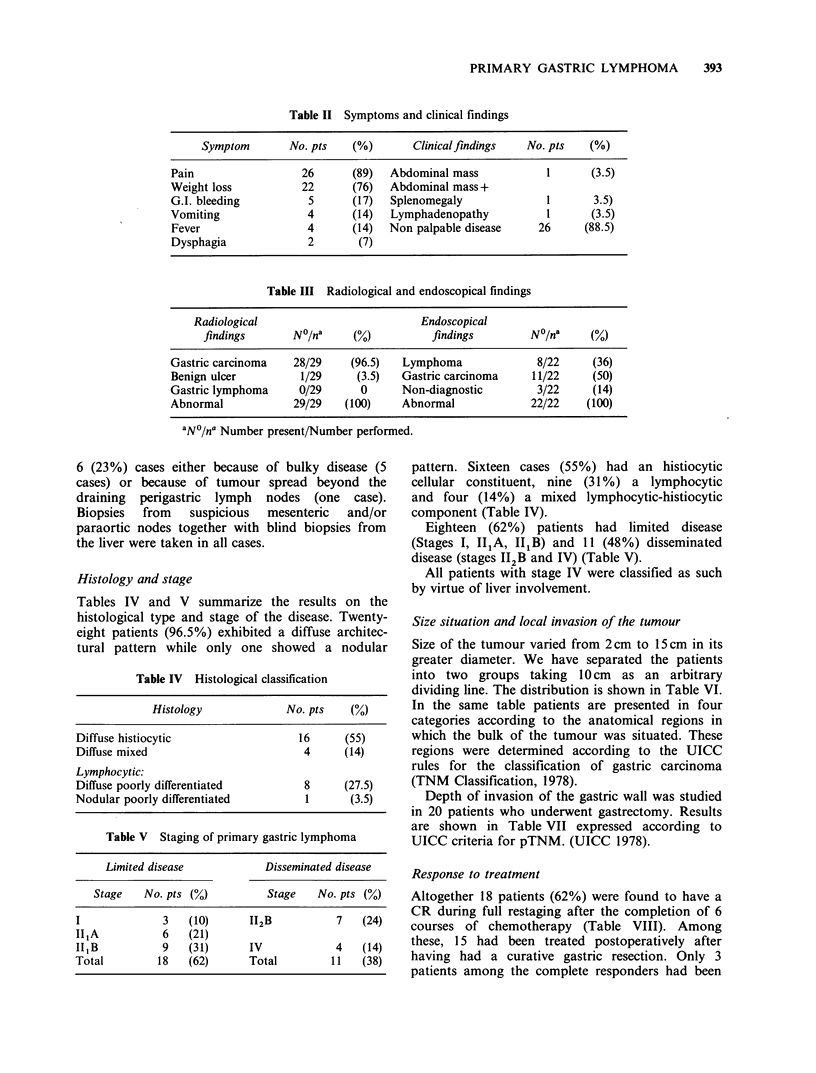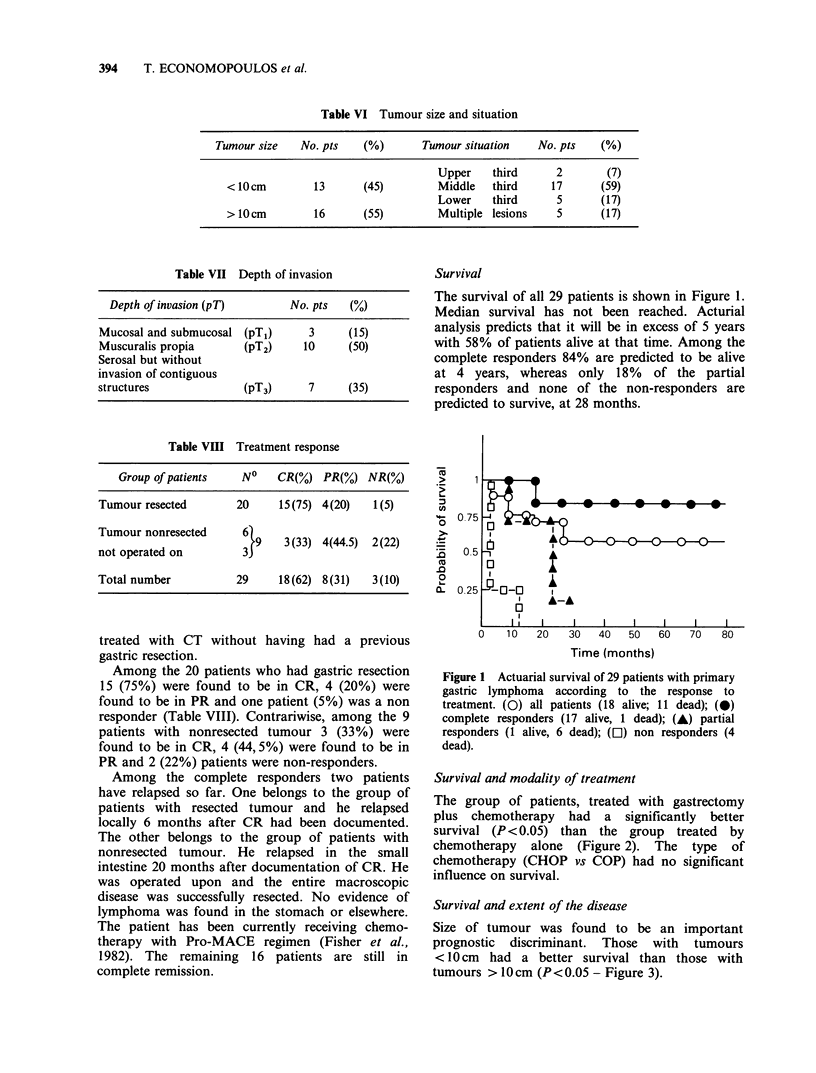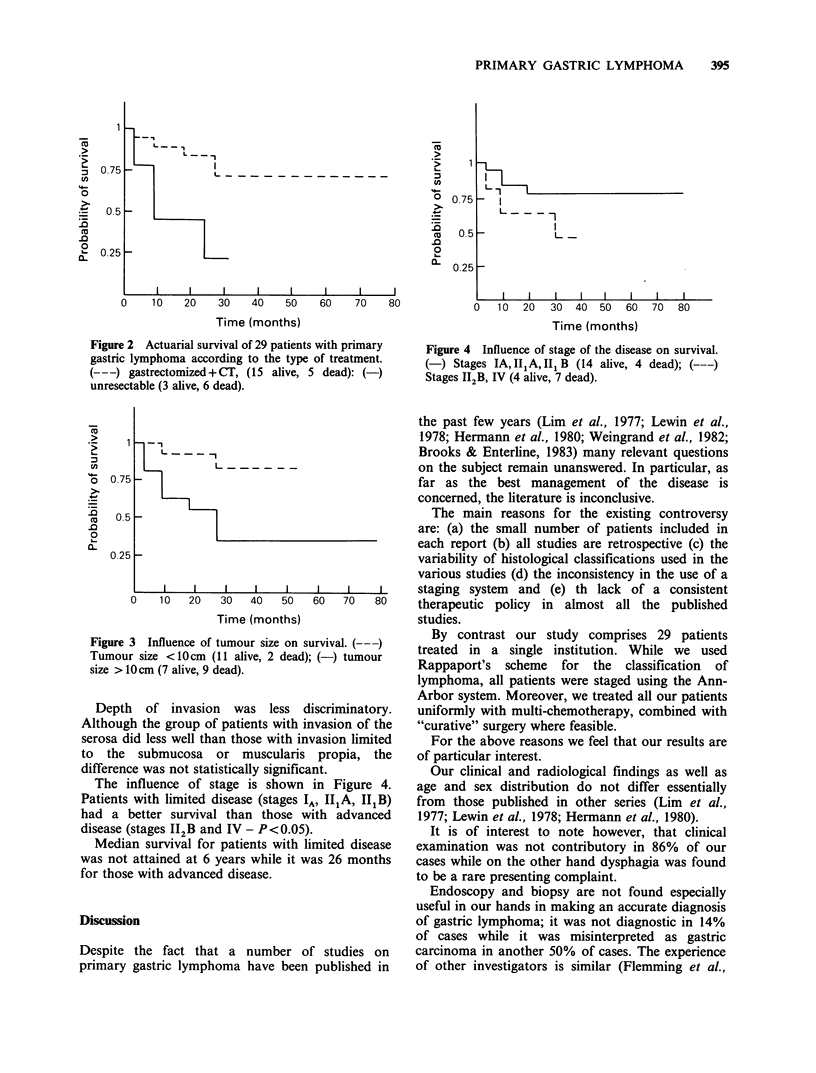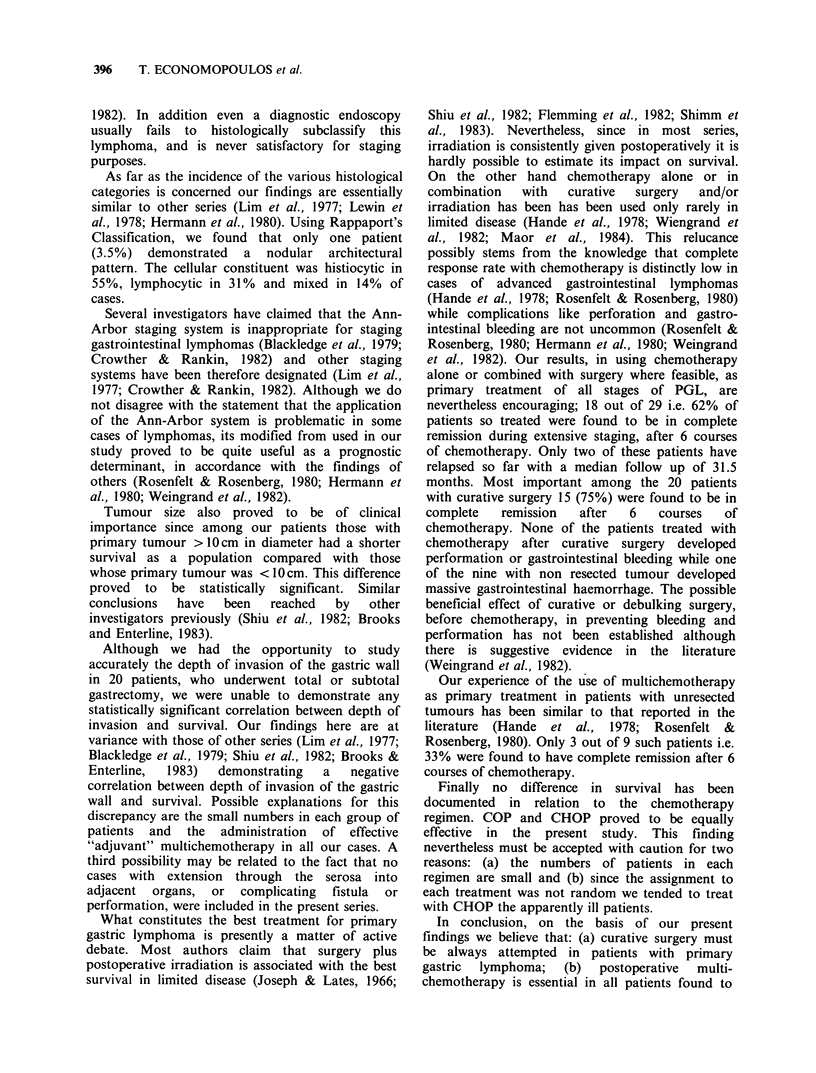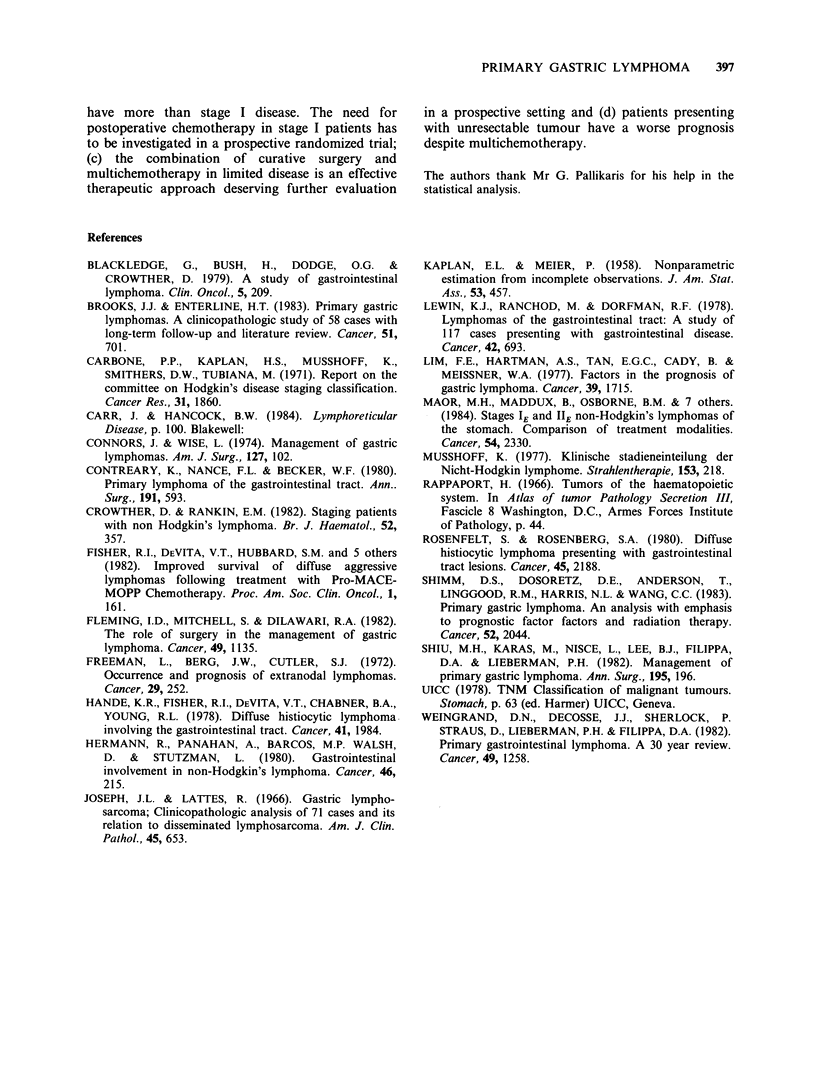# Primary gastric lymphoma--the experience of a general hospital.

**DOI:** 10.1038/bjc.1985.206

**Published:** 1985-09

**Authors:** T. Economopoulos, C. Alexopoulos, N. Stathakis, S. Styloyannis, J. Dervenoulas, S. Tsousis, S. Raptis

## Abstract

We analysed 29 consecutive cases of primary gastric lymphoma (20 men and 9 women) treated in our unit between January 1977 and May 1983. Median age was 55 years. Abdominal pain and weight loss were the main presenting symptoms while there was no palpable disease in the majority of cases. Upper gastrointestinal radiology was abnormal, but not diagnostic, in all cases. Endoscopy with multiple biopsies was performed in 22 cases; carcinoma was diagnosed in 11, lymphoma in 8 while no diagnosis was made in 3 cases. Twenty six patients underwent laparotomy. Gastrectomy was performed in twenty while the tumour was unresectable in six. Histology was reported as diffuse in 28 cases (16 histiocytic, 8 lymphocytic and 4 mixed) and nodular (lymphocytic) in one. All our patients received multichemotherapy. Complete remission after 6 courses was documented in 18 patients (62%). Neither perforation nor gastrointestinal bleeding was a problem in our series. Eighty four per cent complete responders are predicted to be alive at 4 years. Advanced stage (II2B and IV) and tumour size greater than 10 cm adversely influenced survival. We suggest that in limited primary gastric lymphoma an attempt at 'curative' surgery combined with multichemotherapy currently gives very promising results.


*Br. J. Cancer* (1985), **52**, 391–397

### Primary gastric lymphoma – The experience of a General Hospital

T. Economopoulos, C. Alexopoulos, N. Stathakis, S. Styloyannis, J. Dervenoulas, S. Tsousis & S. Raptis

*Second Department of Internal Medicine-Propaedeutic, University of Athens, Evangelismos Hospital, Athens, Greece.*

**Summary** We analysed 29 consecutive cases of primary gastric lymphoma (20 men and 9 women) treated in our unit between January 1977 and May 1983. Median age was 55 years. Abdominal pain and weight loss were the main presenting symptoms while there was no palpable disease in the majority of cases. Upper gastrointestinal radiology was abnormal, but not diagnostic, in all cases. Endoscopy with multiple biopsies was performed in 22 cases; carcinoma was diagnosed in 11, lymphoma in 8 while no diagnosis was made in 3 cases.

Twenty six patients underwent laparotomy. Gastrectomy was performed in twenty while the tumour was unresectable in six. Histology was reported as diffuse in 28 cases (16 histiocytic, 8 lymphocytic and 4 mixed) and nodular (lymphocytic) in one.

All our patients received multichemotherapy. Complete remission after 6 courses was documented in 18 patients (62%). Neither perforation nor gastrointestinal bleeding was a problem in our series. Eighty four per cent complete responders are predicted to be alive at 4 years. Advanced stage ($II_2$B and IV) and tumour size >10 cm adversely influenced survival.

We suggest that in limited primary gastric lymphoma an attempt at 'curative' surgery combined with multichemotherapy currently gives very promising results.

Primary gastric lymphoma (PGL) represents a relatively uncommon gastric neoplasm accounting for ~5% of all gastric cancers (Conors & Wise, 1974; Contreary *et al.*, 1980). However stomach is the most common site of extranodal non Hodgkin's lymphomas (Freeman *et al.*, 1972).

The best treatment for PGL remains uncertain. Although many of the reported series (Fleming *et al.*, 1982; Siu *et al.*, 1982; Shimm *et al.*, 1983) demonstrate that surgical resection with post-operative irradiation is the most effective treatment, the role of chemotherapy as an initial treatment or postoperatively remains to be clarified.

In this article we report our experience with PGL with reference to histology, prognostic factors and the results of combination chemotherapy with or without gastric resection.

### Materials and methods

### *Patients*

By definition patients with PGL have disease confined to stomach or stomach and its draining nodes without evidence of dissemination and with normal peripheral blood at the time of diagnosis (Carr & Hancock, 1984). Applying these criteria we were able to identify a total of 29 patients diagnosed and treated at the Haematology–Oncology section of the Second Department of Internal Medicine, Propaedeutic, Evangelismos Hospital, Athens University, between January 1977 and May 1983.

One patient had a palpable node in the left supraclavicular fossa at presentation but since extensive staging procedures failed to demonstrate disease elsewhere, stomach apart, this patient was not excluded from the study.

The slides of the initial histology were reviewed in all cases, the diagnosis of non-Hodgkin's lymphoma was verified and the cases were finally classified according to the modified Rappaport Classification (1966).

Median follow up of the 29 patients was 29 months with a minimum of 18 months (range 18–79 months).

### *Staging*

All patients were extensively staged on the basis of clinical examination, surgical or endoscopial findings, X-rays of the chest and upper gastro-intestinal (GI) tract, full blood count, liver function tests, bone marrow biopsy, liver biopsy, lymphogram, CT-scan of the abdomen and bone scan.

They were classified according to the Ann-Arbor System (Carbone *et al.*, 1971) with the only modification that stage II disease was further subdivided to stage $II_1$ for those with perigastric or mesenteric nodal involvement and stage $II_2$ for

Correspondence: T. Economopoulos.

Received 26 February 1985; and in revised form 23 May 1985.

© The Macmillan Press Ltd., 1985

those with involvement of the regional but not confluent (paraortic or parailiac) lymph nodes (Musshof, 1977).

*Chemotherapy*

The chemotherapeutic regimens used were either a combination of cyclophosphamide, vinscristine and prednisone (COP) or a combination of all the above drugs plus adriamycin (CHOP). Details of the two regimens are given in Table I. Fifteen patients (51,5%) received COP and 14 patients (48,5%) the CHOP regimen.

*Assessment and definition of response*

All patients without evidence of disease progression during chemotherapy were fully restaged after the completion of 6 courses of treatment. Restaging included clinical examination, full blood count, liver function tests, chest-X-ray, X-rays of the upper GI tract, endoscopy and CT scan of the abdomen. Liver biopsy was repeated at the end of the six courses only if the liver was initially involved. Patients with complete disappearance of disease and no abnormal findings at endoscopial biopsies and liver biopsy were designated as being in complete remission (CR).

Patients with no clinically evident disease, but with laboratory evidence of residual disease and/or abnormal liver biopsy were designated as partial responders (PR). Patients with progressive disease were designated as non-responders (NR).

*Treatment policy*

Twenty six patients (89%) underwent exploratory laparotomy, while 3 (11%) patients proceeded to chemotherapy after a diagnosis of lymphoma on the basis of endoscopical biopsy had been made.

All patients were given combination chemotherapy (CT). A total of 6 courses were given in all responding patients and then full restaging was performed (*supra vide*). Patients in documented CR discontinued CT and they were closely followed up. Patients in PR were given another 3 courses of the same regimen. At this time, if CR was documented, chemotherapy was discontinued while patients still in the PR were given an alternative regimen at the discretion of the physician. No patient in the present series has received irradiation either preoperatively or postoperatively.

*Statistics*

Duration of survival was calculated from the beginning of therapy until death. Survival curves were constructed according to the method of Kaplan–Meier (1958). The significance of differences between the various actuarial survival curves was assessed by the long rank test.

### Results

The patients comprised 20 men and 9 women and the median age of the whole population was 55 years (range, 21–74).

The main clinical manifestations at presentation and the radiological and endoscopical findings are presented in Tables II and III.

Abdominal pain and weight loss were the commonest presenting symptoms. Clinical examination revealed an abdominal mass in two instances (one with splenomegaly) while lymphadenopathy was present in one patient. No disease was palpable in 25 (86%) cases (Table II).

X-ray examination of the upper GI tract was performed in all 29 patients. It was abnormal in all. Radiological findings were interpreted as demonstrating a gastric carcinoma in 28 (96.5%) patients and benign ulcer in one (3.5%).

Endoscopy was performed in 22 cases. Tumour mass and/or various mucosal abnormalities were seen in all, and biopsies were taken from all lesions. Macroscopical findings together with histology were interpreted as showing gastric lymphoma in 8 (36%) cases and gastric carcinoma in 11 (50%) cases, while in the remaining three (14%) cases histology was not diagnostic.

*Surgery*

A total of 26 patients underwent exploratory laparotomy. A gastric resection with curative intent was possible in 20 (77%) cases. It consisted of total gastrectomy in 5 (25%) and subtotal gastrectomy in 15 (75%), depending entirely on the surgeon's discretion. Tumour was considered unresectable in

**Table I** Chemotherapy regimes

| *COP* | | *CHOP* | |
|---|---|---|---|
| Cyclophosphamide | 600 mg m$^{-2}$ days 1 and 8 | Cyclophosphamide | 500 mg days 1 and 8 |
| Vincristine | 2 mg days 1 and 8 | Vincristine | 2 mg days 1 and 8 |
| Prednisone | 30 mg m$^{-2}$ days 1–8 | Adriamycin | 30 mg m$^{-2}$ days 1 and 8 |
| | | Prednisone | 40 mg m$^{-2}$ days 1–8 |

**Table II** Symptoms and clinical findings

| *Symptom* | *No. pts* | (%) | *Clinical findings* | *No. pts* | (%) |
|---|---|---|---|---|---|
| Pain | 26 | (89) | Abdominal mass | 1 | (3.5) |
| Weight loss | 22 | (76) | Abdominal mass + | | |
| G.I. bleeding | 5 | (17) | Splenomegaly | 1 | 3.5) |
| Vomiting | 4 | (14) | Lymphadenopathy | 1 | (3.5) |
| Fever | 4 | (14) | Non palpable disease | 26 | (88.5) |
| Dysphagia | 2 | (7) | | | |

**Table III** Radiological and endoscopical findings

| *Radiological findings* | $N^0/n^a$ | (%) | *Endoscopical findings* | $N^0/n^a$ | (%) |
|---|---|---|---|---|---|
| Gastric carcinoma | 28/29 | (96.5) | Lymphoma | 8/22 | (36) |
| Benign ulcer | 1/29 | (3.5) | Gastric carcinoma | 11/22 | (50) |
| Gastric lymphoma | 0/29 | 0 | Non-diagnostic | 3/22 | (14) |
| Abnormal | 29/29 | (100) | Abnormal | 22/22 | (100) |

[a] $N^0/n^a$ Number present/Number performed.

6 (23%) cases either because of bulky disease (5 cases) or because of tumour spread beyond the draining perigastric lymph nodes (one case). Biopsies from suspicious mesenteric and/or paraortic nodes together with blind biopsies from the liver were taken in all cases.

### *Histology and stage*

Tables IV and V summarize the results on the histological type and stage of the disease. Twenty-eight patients (96.5%) exhibited a diffuse architectural pattern while only one showed a nodular pattern. Sixteen cases (55%) had an histiocytic cellular constituent, nine (31%) a lymphocytic and four (14%) a mixed lymphocytic-histiocytic component (Table IV).

**Table IV** Histological classification

| *Histology* | *No. pts* | (%) |
|---|---|---|
| Diffuse histiocytic | 16 | (55) |
| Diffuse mixed | 4 | (14) |
| *Lymphocytic:* | | |
| Diffuse poorly differentiated | 8 | (27.5) |
| Nodular poorly differentiated | 1 | (3.5) |

**Table V** Staging of primary gastric lymphoma

| *Limited disease* | | | *Disseminated disease* | | |
|---|---|---|---|---|---|
| *Stage* | *No. pts* | (%) | *Stage* | *No. pts* | (%) |
| I | 3 | (10) | $II_2B$ | 7 | (24) |
| $II_1A$ | 6 | (21) | | | |
| $II_1B$ | 9 | (31) | IV | 4 | (14) |
| Total | 18 | (62) | Total | 11 | (38) |

Eighteen (62%) patients had limited disease (Stages I, $II_1A$, $II_1B$) and 11 (48%) disseminated disease (stages $II_2B$ and IV) (Table V).

All patients with stage IV were classified as such by virtue of liver involvement.

### *Size situation and local invasion of the tumour*

Size of the tumour varied from 2 cm to 15 cm in its greater diameter. We have separated the patients into two groups taking 10 cm as an arbitrary dividing line. The distribution is shown in Table VI. In the same table patients are presented in four categories according to the anatomical regions in which the bulk of the tumour was situated. These regions were determined according to the UICC rules for the classification of gastric carcinoma (TNM Classification, 1978).

Depth of invasion of the gastric wall was studied in 20 patients who underwent gastrectomy. Results are shown in Table VII expressed according to UICC criteria for pTNM. (UICC 1978).

### *Response to treatment*

Altogether 18 patients (62%) were found to have a CR during full restaging after the completion of 6 courses of chemotherapy (Table VIII). Among these, 15 had been treated postoperatively after having had a curative gastric resection. Only 3 patients among the complete responders had been

**Table VI** Tumour size and situation

| *Tumour size* | *No. pts* | (%) | *Tumour situation* | *No. pts* | (%) |
|---|---|---|---|---|---|
| | | | Upper third | 2 | (7) |
| <10 cm | 13 | (45) | Middle third | 17 | (59) |
| | | | Lower third | 5 | (17) |
| >10 cm | 16 | (55) | Multiple lesions | 5 | (17) |

**Table VII** Depth of invasion

| *Depth of invasion (pT)* | | *No. pts* | (%) |
|---|---|---|---|
| Mucosal and submucosal | ($pT_1$) | 3 | (15) |
| Muscuralis propia | ($pT_2$) | 10 | (50) |
| Serosal but without invasion of contiguous structures | ($pT_3$) | 7 | (35) |

**Table VIII** Treatment response

| *Group of patients* | $N^0$ | | *CR(%)* | *PR(%)* | *NR(%)* |
|---|---|---|---|---|---|
| Tumour resected | 20 | | 15(75) | 4(20) | 1(5) |
| Tumour nonresected | 6 | 9 | 3(33) | 4(44.5) | 2(22) |
| not operated on | 3 | | | | |
| Total number | 29 | | 18(62) | 8(31) | 3(10) |

treated with CT without having had a previous gastric resection.

Among the 20 patients who had gastric resection 15 (75%) were found to be in CR, 4 (20%) were found to be in PR and one patient (5%) was a non responder (Table VIII). Contrariwise, among the 9 patients with nonresected tumour 3 (33%) were found to be in CR, 4 (44,5%) were found to be in PR and 2 (22%) patients were non-responders.

Among the complete responders two patients have relapsed so far. One belongs to the group of patients with resected tumour and he relapsed locally 6 months after CR had been documented. The other belongs to the group of patients with nonresected tumour. He relapsed in the small intestine 20 months after documentation of CR. He was operated upon and the entire macroscopic disease was successfully resected. No evidence of lymphoma was found in the stomach or elsewhere. The patient has been currently receiving chemotherapy with Pro-MACE regimen (Fisher *et al.*, 1982). The remaining 16 patients are still in complete remission.

### *Survival*

The survival of all 29 patients is shown in Figure 1. Median survival has not been reached. Acturial analysis predicts that it will be in excess of 5 years with 58% of patients alive at that time. Among the complete responders 84% are predicted to be alive at 4 years, whereas only 18% of the partial responders and none of the non-responders are predicted to survive, at 28 months.

**Figure 1** Actuarial survival of 29 patients with primary gastric lymphoma according to the response to treatment. (○) all patients (18 alive; 11 dead); (●) complete responders (17 alive, 1 dead); (▲) partial responders (1 alive, 6 dead); (□) non responders (4 dead).

### *Survival and modality of treatment*

The group of patients, treated with gastrectomy plus chemotherapy had a significantly better survival ($P<0.05$) than the group treated by chemotherapy alone (Figure 2). The type of chemotherapy (CHOP *vs* COP) had no significant influence on survival.

### *Survival and extent of the disease*

Size of tumour was found to be an important prognostic discriminant. Those with tumours <10 cm had a better survival than those with tumours >10 cm ($P<0.05$ – Figure 3).

**Figure 2** Actuarial survival of 29 patients with primary gastric lymphoma according to the type of treatment. (---) gastrectomized+CT, (15 alive, 5 dead): (—) unresectable (3 alive, 6 dead).

**Figure 3** Influence of tumour size on survival. (---) Tumour size <10 cm (11 alive, 2 dead); (—) tumour size >10 cm (7 alive, 9 dead).

Depth of invasion was less discriminatory. Although the group of patients with invasion of the serosa did less well than those with invasion limited to the submucosa or muscularis propia, the difference was not statistically significant.

The influence of stage is shown in Figure 4. Patients with limited disease (stages $I_A$, $II_1A$, $II_1B$) had a better survival than those with advanced disease (stages $II_2B$ and IV – $P<0.05$).

Median survival for patients with limited disease was not attained at 6 years while it was 26 months for those with advanced disease.

### Discussion

Despite the fact that a number of studies on primary gastric lymphoma have been published in

**Figure 4** Influence of stage of the disease on survival. (—) Stages IA, $II_1A$, $II_1B$ (14 alive, 4 dead); (---) Stages $II_2B$, IV (4 alive, 7 dead).

the past few years (Lim *et al.*, 1977; Lewin *et al.*, 1978; Hermann *et al.*, 1980; Weingrand *et al.*, 1982; Brooks & Enterline, 1983) many relevant questions on the subject remain unanswered. In particular, as far as the best management of the disease is concerned, the literature is inconclusive.

The main reasons for the existing controversy are: (a) the small number of patients included in each report (b) all studies are retrospective (c) the variability of histological classifications used in the various studies (d) the inconsistency in the use of a staging system and (e) th lack of a consistent therapeutic policy in almost all the published studies.

By contrast our study comprises 29 patients treated in a single institution. While we used Rappaport's scheme for the classification of lymphoma, all patients were staged using the Ann-Arbor system. Moreover, we treated all our patients uniformly with multi-chemotherapy, combined with "curative" surgery where feasible.

For the above reasons we feel that our results are of particular interest.

Our clinical and radiological findings as well as age and sex distribution do not differ essentially from those published in other series (Lim *et al.*, 1977; Lewin *et al.*, 1978; Hermann *et al.*, 1980).

It is of interest to note however, that clinical examination was not contributory in 86% of our cases while on the other hand dysphagia was found to be a rare presenting complaint.

Endoscopy and biopsy are not found especially useful in our hands in making an accurate diagnosis of gastric lymphoma; it was not diagnostic in 14% of cases while it was misinterpreted as gastric carcinoma in another 50% of cases. The experience of other investigators is similar (Flemming *et al.*,

1982). In addition even a diagnostic endoscopy usually fails to histologically subclassify this lymphoma, and is never satisfactory for staging purposes.

As far as the incidence of the various histological categories is concerned our findings are essentially similar to other series (Lim *et al.*, 1977; Lewin *et al.*, 1978; Hermann *et al.*, 1980). Using Rappaport's Classification, we found that only one patient (3.5%) demonstrated a nodular architectural pattern. The cellular constituent was histiocytic in 55%, lymphocytic in 31% and mixed in 14% of cases.

Several investigators have claimed that the Ann-Arbor staging system is inappropriate for staging gastrointestinal lymphomas (Blackledge *et al.*, 1979; Crowther & Rankin, 1982) and other staging systems have been therefore designated (Lim *et al.*, 1977; Crowther & Rankin, 1982). Although we do not disagree with the statement that the application of the Ann-Arbor system is problematic in some cases of lymphomas, its modified from used in our study proved to be quite useful as a prognostic determinant, in accordance with the findings of others (Rosenfelt & Rosenberg, 1980; Hermann *et al.*, 1980; Weingrand *et al.*, 1982).

Tumour size also proved to be of clinical importance since among our patients those with primary tumour >10 cm in diameter had a shorter survival as a population compared with those whose primary tumour was <10 cm. This difference proved to be statistically significant. Similar conclusions have been reached by other investigators previously (Shiu *et al.*, 1982; Brooks and Enterline, 1983).

Although we had the opportunity to study accurately the depth of invasion of the gastric wall in 20 patients, who underwent total or subtotal gastrectomy, we were unable to demonstrate any statistically significant correlation between depth of invasion and survival. Our findings here are at variance with those of other series (Lim *et al.*, 1977; Blackledge *et al.*, 1979; Shiu *et al.*, 1982; Brooks & Enterline, 1983) demonstrating a negative correlation between depth of invasion of the gastric wall and survival. Possible explanations for this discrepancy are the small numbers in each group of patients and the administration of effective "adjuvant" multichemotherapy in all our cases. A third possibility may be related to the fact that no cases with extension through the serosa into adjacent organs, or complicating fistula or performation, were included in the present series.

What constitutes the best treatment for primary gastric lymphoma is presently a matter of active debate. Most authors claim that surgery plus postoperative irradiation is associated with the best survival in limited disease (Joseph & Lates, 1966; Shiu *et al.*, 1982; Flemming *et al.*, 1982; Shimm *et al.*, 1983). Nevertheless, since in most series, irradiation is consistently given postoperatively it is hardly possible to estimate its impact on survival. On the other hand chemotherapy alone or in combination with curative surgery and/or irradiation has been has been used only rarely in limited disease (Hande *et al.*, 1978; Wiengrand *et al.*, 1982; Maor *et al.*, 1984). This relucance possibly stems from the knowledge that complete response rate with chemotherapy is distinctly low in cases of advanced gastrointestinal lymphomas (Hande *et al.*, 1978; Rosenfelt & Rosenberg, 1980) while complications like perforation and gastro-intestinal bleeding are not uncommon (Rosenfelt & Rosenberg, 1980; Hermann *et al.*, 1980; Weingrand *et al.*, 1982). Our results, in using chemotherapy alone or combined with surgery where feasible, as primary treatment of all stages of PGL, are nevertheless encouraging; 18 out of 29 i.e. 62% of patients so treated were found to be in complete remission during extensive staging, after 6 courses of chemotherapy. Only two of these patients have relapsed so far with a median follow up of 31.5 months. Most important among the 20 patients with curative surgery 15 (75%) were found to be in complete remission after 6 courses of chemotherapy. None of the patients treated with chemotherapy after curative surgery developed performation or gastrointestinal bleeding while one of the nine with non resected tumour developed massive gastrointestinal haemorrhage. The possible beneficial effect of curative or debulking surgery, before chemotherapy, in preventing bleeding and performation has not been established although there is suggestive evidence in the literature (Weingrand *et al.*, 1982).

Our experience of the use of multichemotherapy as primary treatment in patients with unresected tumours has been similar to that reported in the literature (Hande *et al.*, 1978; Rosenfelt & Rosenberg, 1980). Only 3 out of 9 such patients i.e. 33% were found to have complete remission after 6 courses of chemotherapy.

Finally no difference in survival has been documented in relation to the chemotherapy regimen. COP and CHOP proved to be equally effective in the present study. This finding nevertheless must be accepted with caution for two reasons: (a) the numbers of patients in each regimen are small and (b) since the assignment to each treatment was not random we tended to treat with CHOP the apparently ill patients.

In conclusion, on the basis of our present findings we believe that: (a) curative surgery must be always attempted in patients with primary gastric lymphoma; (b) postoperative multi-chemotherapy is essential in all patients found to have more than stage I disease. The need for postoperative chemotherapy in stage I patients has to be investigated in a prospective randomized trial; (c) the combination of curative surgery and multichemotherapy in limited disease is an effective therapeutic approach deserving further evaluation in a prospective setting and (d) patients presenting with unresectable tumour have a worse prognosis despite multichemotherapy.

The authors thank Mr G. Pallikaris for his help in the statistical analysis.

**References**

BLACKLEDGE, G., BUSH, H., DODGE, O.G. & CROWTHER, D. 1979). A study of gastrointestinal lymphoma. *Clin. Oncol.*, **5**, 209.

BROOKS, J.J. & ENTERLINE, H.T. (1983). Primary gastric lymphomas. A clinicopathologic study of 58 cases with long-term follow-up and literature review. *Cancer*, **51**, 701.

CARBONE, P.P., KAPLAN, H.S., MUSSHOFF, K., SMITHERS, D.W., TUBIANA, M. (1971). Report on the committee on Hodgkin's disease staging classification. *Cancer Res.*, **31**, 1860.

CARR, J. & HANCOCK, B.W. (1984). *Lymphoreticular Disease*, p. 100. Blakewell:

CONNORS, J. & WISE, L. (1974). Management of gastric lymphomas. *Am. J. Surg.*, **127**, 102.

CONTREARY, K., NANCE, F.L. & BECKER, W.F. (1980). Primary lymphoma of the gastrointestinal tract. *Ann.. Surg.*, **191**, 593.

CROWTHER, D. & RANKIN, E.M. (1982). Staging patients with non Hodgkin's lymphoma. *Br. J. Haematol.*, **52**, 357.

FISHER, R.I., DEVITA, V.T., HUBBARD, S.M. and 5 others (1982). Improved survival of diffuse aggressive lymphomas following treatment with Pro-MACE-MOPP Chemotherapy. *Proc. Am. Soc. Clin. Oncol.*, **1**, 161.

FLEMING, I.D., MITCHELL, S. & DILAWARI, R.A. (1982). The role of surgery in the management of gastric lymphoma. *Cancer*, **49**, 1135.

FREEMAN, L., BERG, J.W., CUTLER, S.J. (1972). Occurrence and prognosis of extranodal lymphomas. *Cancer*, **29**, 252.

HANDE, K.R., FISHER, R.I., DEVITA, V.T., CHABNER, B.A., YOUNG, R.L. (1978). Diffuse histiocytic lymphoma involving the gastrointestinal tract. *Cancer*, **41**, 1984.

HERMANN, R., PANAHAN, A., BARCOS, M.P. WALSH, D. & STUTZMAN, L. (1980). Gastrointestinal involvement in non-Hodgkin's lymphoma. *Cancer*, **46**, 215.

JOSEPH, J.L. & LATTES, R. (1966). Gastric lymphosarcoma; Clinicopathologic analysis of 71 cases and its relation to disseminated lymphosarcoma. *Am. J. Clin. Pathol.*, **45**, 653.

KAPLAN, E.L. & MEIER, P. (1958). Nonparametric estimation from incomplete observations. *J. Am. Stat. Ass.*, **53**, 457.

LEWIN, K.J., RANCHOD, M. & DORFMAN, R.F. (1978). Lymphomas of the gastrointestinal tract: A study of 117 cases presenting with gastrointestinal disease. *Cancer*, **42**, 693.

LIM, F.E., HARTMAN, A.S., TAN, E.G.C., CADY, B. & MEISSNER, W.A. (1977). Factors in the prognosis of gastric lymphoma. *Cancer*, **39**, 1715.

MAOR, M.H., MADDUX, B., OSBORNE, B.M. & 7 others. (1984). Stages $I_E$ and $II_E$ non-Hodgkin's lymphomas of the stomach. Comparison of treatment modalities. *Cancer*, **54**, 2330.

MUSSHOFF, K. (1977). Klinische stadieneinteilung der Nicht-Hodgkin lymphome. *Strahlentherapie*, **153**, 218.

RAPPAPORT, H. (1966). Tumors of the haematopoietic system. In *Atlas of tumor Pathology Secretion III*, Fascicle 8 Washington, D.C., Armes Forces Institute of Pathology, p. 44.

ROSENFELT, S. & ROSENBERG, S.A. (1980). Diffuse histiocytic lymphoma presenting with gastrointestinal tract lesions. *Cancer*, **45**, 2188.

SHIMM, D.S., DOSORETZ, D.E., ANDERSON, T., LINGGOOD, R.M., HARRIS, N.L. & WANG, C.C. (1983). Primary gastric lymphoma. An analysis with emphasis to prognostic factor factors and radiation therapy. *Cancer*, **52**, 2044.

SHIU, M.H., KARAS, M., NISCE, L., LEE, B.J., FILIPPA, D.A. & LIEBERMAN, P.H. (1982). Management of primary gastric lymphoma. *Ann. Surg.*, **195**, 196.

UICC (1978). TNM Classification of malignant tumours. *Stomach*, p. 63 (ed. Harmer) UICC, Geneva.

WEINGRAND, D.N., DECOSSE, J.J., SHERLOCK, P. STRAUS, D., LIEBERMAN, P.H. & FILIPPA, D.A. (1982). Primary gastrointestinal lymphoma. A 30 year review. *Cancer*, **49**, 1258.